# Stereotactic Body Radiation Therapy for Prostate Cancer Patients with Old Age or Medical Comorbidity: A 5-year Follow-Up of an Investigational Study

**DOI:** 10.1097/MD.0000000000000290

**Published:** 2014-12-02

**Authors:** Sea-Won Lee, Hong Seok Jang, Jong Hoon Lee, Sung Hwan Kim, Sei Chul Yoon

**Affiliations:** From the Department of Radiation Oncology, St. Vincent's Hospital, The Catholic University of Korea, College of Medicine, Seoul, Republic of Korea (S-WL, JHL, SHK); Department of Radiation Oncology, Seoul St. Mary's Hospital, The Catholic University of Korea, College of Medicine, Seoul, Republic of Korea (HSJ); and Department of Radiation Oncology, Bucheon St. Mary's Hospital, The Catholic University of Korea, College of Medicine, Seoul, Republic of Korea (SCY)

## Abstract

We evaluated 5-year follow-up of stereotactic body radiation therapy (SBRT) with Cyberknife for prostate cancer patients.

Forty-five men with prostate adenocarcinoma who received SBRT using Cyberknife from May 2006 to November 2012 were enrolled in this study. They were prostate cancer patients with old age and medical comorbidities who received a total of 36 Gy to the prostate in 5 fractions with either everyday or every other day schedule. Prostate-specific antigen (PSA) levels at initial diagnosis and after radiation were traced. Primary endpoints were biochemical relapse-free survival (bRFS), progression-free survival (PFS), and overall survival (OS). The definition of biochemical relapse was a PSA level of nadir + 2 ng/mL. Progression was defined as biochemically or clinically detected disease and the start of salvage therapy.

After median follow-up of 63 months, the 5-year bRFS for all patients was estimated at 89.7%. The 5-year PFS was estimated at 71%. Four cases of biochemical relapse were observed, including two patients who experienced locoregional failure and one patient who had distant metastasis with biochemical relapse. The 5-year OS was estimated at 94.3%. There were five deaths, all of which were unrelated to prostate cancer. There was no grade 3 or higher acute complication. Grade 3 or higher late urinary toxicity was reported in 2 (4.4%) of 45 patients.

The 5-year survival and toxicity outcome of SBRT using Cyberknife on prostate cancer patients with old age or comorbidities were favorable and safe in an investigational study.

## INTRODUCTION

Prostate cancer is well known for its low α/β ratio, which is probably lower than that of the normal tissues.^1,2^ Although the argument on how low the α/β ratio remains, the therapeutic advantage of large fraction size for prostate cancer is indisputable. Since the early applications of hypofractionation for prostate began in the 1960s, numerous trials have tested its feasibility and effectiveness in clinical settings.^3^ Currently, stereotactic body radiotherapy (SBRT) using image-guided and intensity-modulated radiation therapy to deliver high doses on prostate but spare normal organs at the same time is being widely investigated.

Several initial outcomes of 2 to 3 years after Cyberknife (Accuray, Sunnyvale, CA) SBRT have been published to date.^4,5^ Most of them are still under investigation. Few published studies have been followed up to 5 years. Freeman and King^4^ presented the first 5-year outcomes of an ongoing phase II clinical trial with 41 patients who received SBRT of 35 to 36.25 Gy in 5 fractions using Cyberknife. In their report, biochemical relapse-free survival (bRFS) was 93%. More recently, Katz et al^5^ reported the 5-year results of a larger subset of patients who received SBRT. However, these studies were conducted on organ-confined disease, and the underlying medical conditions of their patient population were not specified.

As a result of dedication to treatment individualization, radiation oncologists were often consulted on SBRT in prostate cancer patients with old age and comorbidities who were inapt for surgery. This population of patients who were contraindicated for radical surgery tended to have more underlying diseases than prostate cancer patients with resectable disease. Clinical studies of SBRT in prostate cancer patients with old age and comorbidities are scarce and preliminary in Asian countries.^6^ Thus, we performed an investigational study on 5-year follow-up and toxicity of SBRT using Cyberknife in Asian prostate cancer patients with old age and medical comorbidities.

## MATERIALS AND METHODS

### Patients

A total of 45 men who had been histologically diagnosed with prostate adenocarcinoma were treated with SBRT using Cyberknife from May 2006 to November 2012. They were histologically confirmed prostate cancer patients with old age and/or medical comorbidities who were unsuitable for radical prostatectomy. Patients with clinical T2–3 disease were included. Therefore, this study was not limited to organ-confined or early-staged disease. Exclusion criteria were clinical node positivity and distant metastasis. This study was approved by the institutional review board of our institution.

Clinical staging work-up included digital rectal examination, complete blood count, level of prostate-specific antigen (PSA), chest and abdomen CT, pelvic MRI, and bone scan before radiotherapy.

### Treatment

The Cyberknife was selected as the modality for SBRT, hence delivering image-guided and intensity-modulated radiation therapy. Three to four gold fiducials were inserted in the prostate gland using trans-rectal ultrasonography. Patients were supinely positioned with arms on the chest. They were fixed using vacuumed cushion. Simulation computed tomography (CT) was scanned with contrast-enhancement in 1.5 mm thickness.

Regions of interest (including seminal vesicles, rectum, bladder, and femoral heads) were anatomically contoured. Prostate and involved seminal vesicle was the gross tumor volume (GTV). The planning target volume (PTV) was created by expanding GTV with 5 mm margin in all directions except for the posterior side, for which the margin was reduced to 2.5 mm. All patients were homogeneously planned so that the isodose line covered >95% of PTV as well as satisfying the limitation of rectal D50% <50% (i.e. <50% of the rectal volume received 50% of the prescribed dose) and D100% <5%. A pair of orthogonal X-ray imaging system enabled real-time image guidance both intrafraction and interfraction wise to achieve accurate and precise beam delivery.

Patients received a total of 36 Gy to the PTV in 5 fractions. Supposing the α/β ratio of prostate is 1.5, this total dose is biologically equivalent to 89.5 Gy in 2 Gy fractions.^6^ The hypofractionated radiation was given either every day or every other day schedule. Seventeen (37.8%) patients who received SBRT on the first 3 years were treated every day. The remaining (62.2%) patients followed the schedule of every other day.

### Evaluation and Statistical Analysis

Patients had appointments after SBRT for clinic visit in 1 week, 1 month, and then every 3 months thereafter for at least the first 5 years. PSA was followed up at 3-month interval. Patients who reached and maintained their PSA nadir were allowed 6-month visits. Patients showing PSA levels of rising tendency with suspicion of disease progression were examined using imaging techniques such as abdomino-pelvic CT, prostate MRI, bone scan, and PET-CT.

Biochemical relapse-free survival (bRFS), progression-free survival (PFS), and overall survival (OS) were primary endpoints of this study. Biochemical relapse was defined as a PSA level of nadir + 2 ng/mL. Progression was defined as biochemically or clinically detected disease or the start of salvage therapy. Radiation toxicity was a secondary endpoint. Acute toxicity was any symptom or sign reported within 90 days after finishing radiotherapy. Any symptom or sign recorded thereafter was defined as late toxicity. The urinary and rectal toxicities were graded according to the Common Terminology Criteria for Adverse Events (CTCAE) Version 4.0. Grade 2 or higher toxicities were recorded. Most patients had underlying benign prostatic diseases with initial symptoms before starting the radiation. Any aggravation or development of new symptoms and signs compared to the baseline was considered radiation-induced.

*T*-test was used to determine the significance of associations between continuous variables. Survival rates were calculated using Kaplan–Meier method. All tests were two-sided. Statistically significant survival difference between two groups was examined using the log-rank test. Cox proportional hazard model was used for multivariable analysis. Statistical significance was considered when *P* value was <0.05.

## RESULTS

The median age of patients was 73 years (range, 50–86 yrs). Thirteen (28.8%) of 45 patients were in high-risk group according to the National Comprehensive Cancer Network Guideline (T3 or Gleason 8–10 or PSA >20 ng/mL). Pathology of eight (13.3%) patients scored Gleason score of ≥8. Six (13.3%) patients had clinical T3 diseases. Thirty-five (77.8%) patients had underlying medical comorbidities such as coronary, cerebrovascular, and obstructive lung disease. Twenty-four (53.3%) patients were found to have >2 underlying medical diseases. Patient characteristics are summarized in Table 1.

**TABLE 1:**
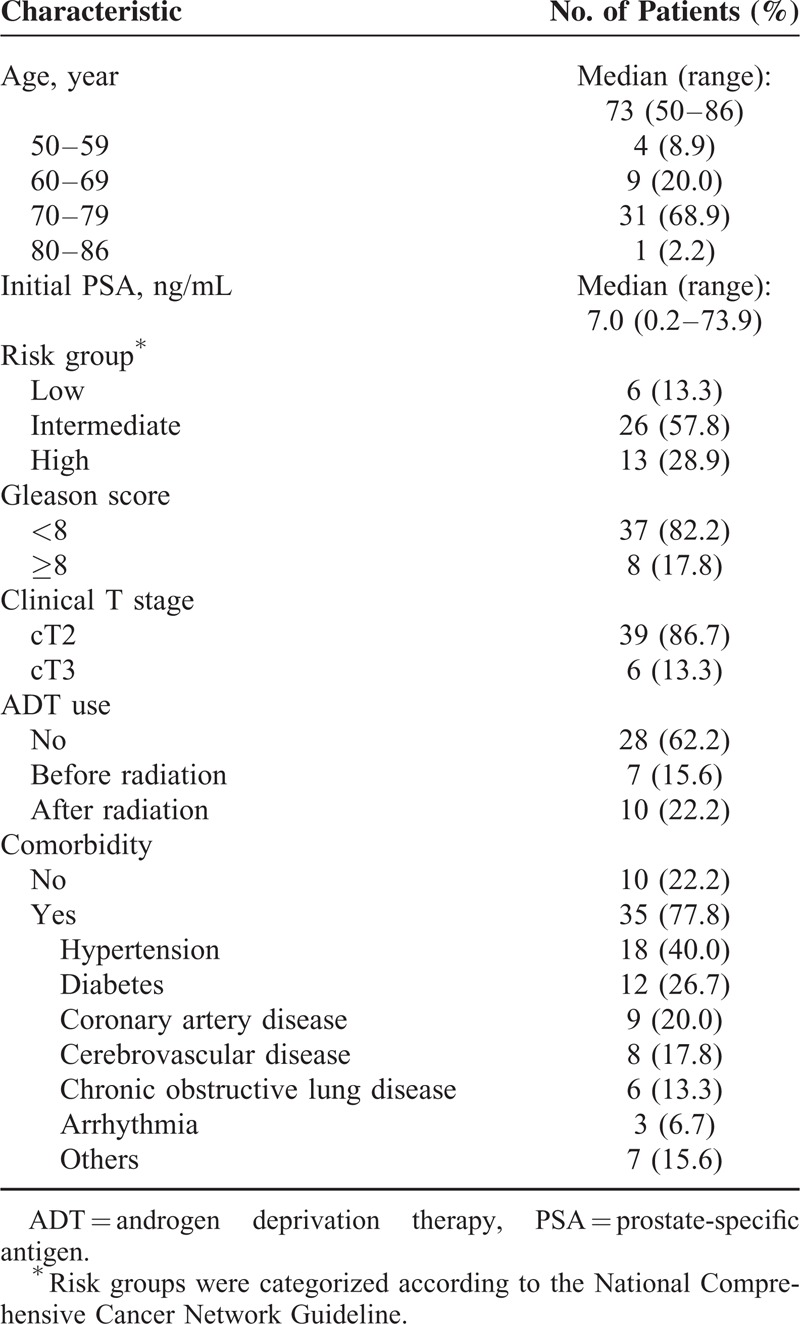
Patient Characteristics (n = 45)

### Biochemical Relapse, Progression, and Survival

**FIGURE 1:**
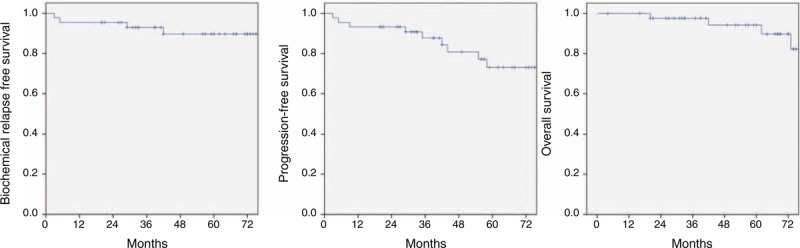
Survival outcomes of the 45 prostate cancer patients who received stereotactic body radiation treatment are shown. The biochemical relapse-free survival at 5 years was 89.7% (A). The progression-free survival at 5 years was 71% (B). The overall survival at 5 years was 94.3% (C).

**FIGURE 2:**
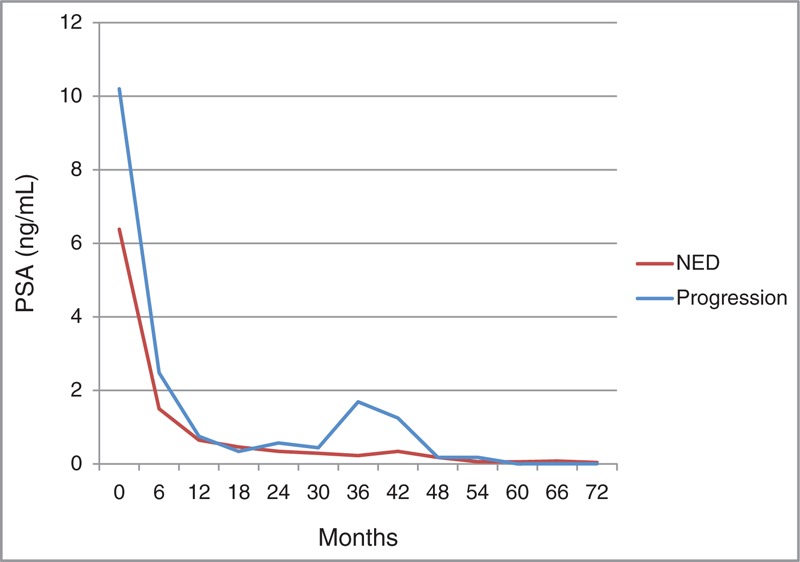
The mean PSA levels acquired every 6 months for patients with NED and patients with progression. PSA = prostate-specific antigen; NED = no evidence of disease.

After median follow-up of 63 months, bRFS, PFS, and OS are depicted in Figure 1. The 5-year bRFS of all patients was estimated at 89.7%. Four cases of biochemical relapse were observed. The mean PSA levels at every 6 months for patients without evidence of disease (NED) and patients with progression were plotted in Figure 2. The initial mean PSA for progressed patients was significantly higher than patients with NED (10.2 vs. 6.3 ng/mL, *P* = 0.04). The median time to progression was 33 months shown in the graph of progressed patients who had rapid rise of PSA between 30 and 36 months. The consequential PSA decrease was observed due to immediate application of salvage treatment including hormonal therapy.

**FIGURE 3:**
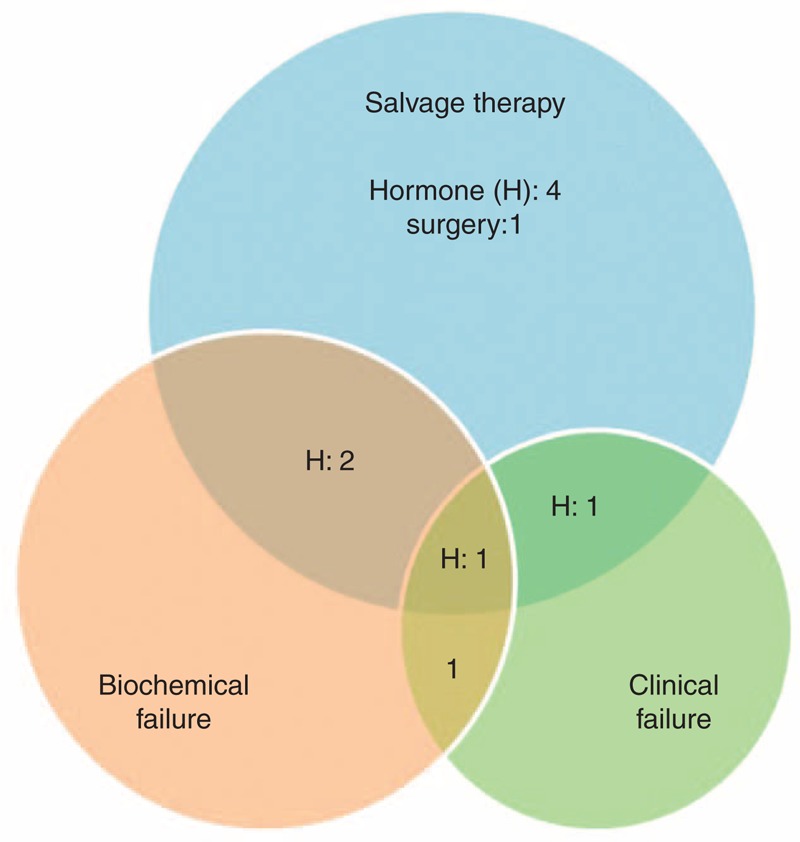
The pattern of progression.

The 5-year PFS was estimated at 71%. Of the four biochemically progressed patients, two experienced locoregional failure, and one had bone metastasis without biochemical relapse. Thus, there were 3 patients with clinical progression. Of the 5 patients who received salvage treatments, four patients were given hormonal therapy and one patient received surgery. The pattern of progression is illustrated in Figure 3.

The 5-year OS was estimated at 94.3%. There were five deaths in total, all of which were unrelated to prostate cancer.

### Risk Factor Analysis for Progression Free Survival

Risk factors for PFS were summarized in Table 2. Based on univariate analysis, patient age, risk group, and clinical T stage did not significantly affect the PFS. However, initial PSA (*P* = 0.04), nadir PSA (*P* = 0.02), and Gleason score (*P* = 0.02) significantly affected the PFS. When these factors were entered into multivariate analysis using a Cox regression model, no factors retained significance as a prognostic factor for the PFS.

**TABLE 2:**
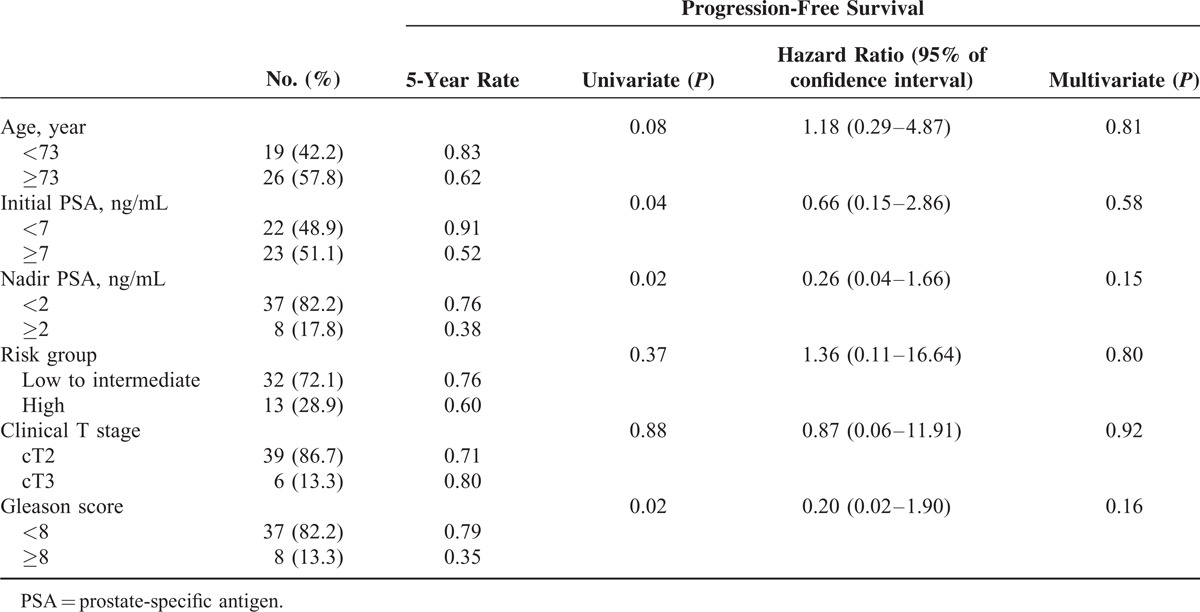
Risk Factor Analysis Affecting Progression-Free Survival

### Toxicity

The rates of acute and late toxicities are summarized in Table 3. There was no grade 3 or higher acute complication. Grade 2 urinary toxicity was observed in two (4.4%) patients. Two (4.4%) patients had grade 2 acute rectal toxicity of transient blood-tinged stool confirmed by colonoscopy. The rectal symptoms were controlled with supportive care. Grade 3 late urinary toxicity was reported in two (4.4%) of 45 patients. Two patients received trans-urethral resection due to the aggravation of underlying benign prostatic hyperplasia. Two patients experienced persistent grade 2 rectal radiation proctitis on colonoscopy 3 months after SBRT.

**TABLE 3:**
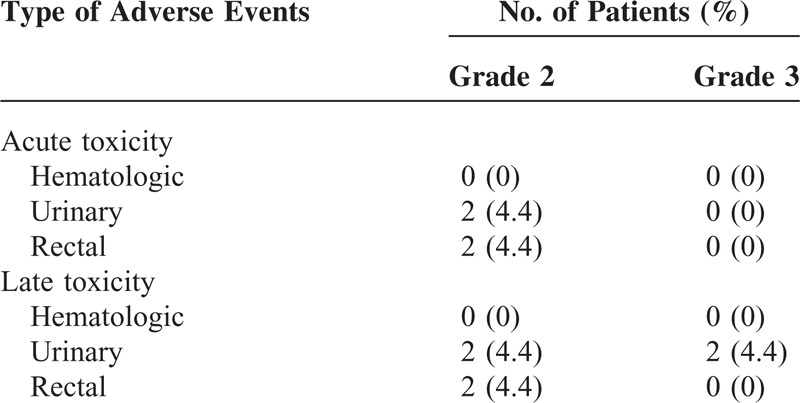
Grade 2 or Higher Adverse Events

## DISCUSSION

The first hypofractionated radiation on prostate which was delivered in the United Kingdom dates back to the early 1960s. The regimen of 36 Gy given over 3 weeks in 5 fractions was devised for purely economic reasons. Nevertheless, with longest history, the 22 years’ experience (1962–1984) proved an efficacy in tumor control and acceptable toxicity in the pre-PSA era using two-dimensional technique.^7^ This so called “extreme” hypofractionation (total 35–50 Gy in 4–6 fractions) was discarded for another decade until the application of high dose-rate brachytherapy in 1990s.

As a general rule, conventional fractionation (1.8–2 Gy per fraction, total 38–45 fractions) has been the mainstay in prostate radiation due to its radiobiologic benefits of redistribution, reoxygenation, and allowing time for repair of sublethal damage in the normal tissues.^8^ However, the supposition that prostate has α/β ratio of 1.5, which is far less compared to the ratio of 3 to 5 for late-responding normal tissues has been suggested in the past few decades.^9^ This means that large fraction size delivered in fewer fractions may increase therapeutic ratio and lower normal tissue damage. The outstanding tumor control and reduced toxicity exhibited by high dose-rate brachytherapy using hypofractionated regimen supports the modern understanding of prostate radiobiology. This radiobiologic advancement has introduced moderate hypofractionation (2.5–3 Gy per fraction up to 20–28 fractions) into practice.

Of diverse hypofractionation regimens currently being developed and tried, doses of ≥35 Gy in 5 fractions have higher normalized total dose at 2 Gy per fraction, leading to better predicted bRFS.^10^ This rationale together with excellent results shown by brachytherapy strongly supports the application of hypofractionated SBRT. This is the background in selecting our dose regimen of 36 Gy given in 5 fractions.

Hypofractionation means high dose delivery with exact targeting. It also means fewer chances for adjustments. These limitations are overcome with technologic and radiophysical progress which has made SBRT possible. SBRT may be given using IMRT by gantry-based linear accelerators or TomoTherapy (Accuray, Sunnyvale, CA) or Cyberknife. Cyberknife was the first SBRT delivery modality tested for prostate. Approximately 10,000 prostate cancer patients have been treated with it since 2003.^8^ The robotic arm of Cyberknife with multiple non-isocentric, non-coplanar pencil beam enables highly conformal plan. Hossain et al^11^ reported the conformality indices for Cyberknife plans were superior to those of other IMRT plans. However, the steep dose gradient of SBRT is greatly influenced by organ movements. The real-time motion tracking ability of Cyberknife considerably reduces the organ movement to submillimeter level. Therefore, we considered Cyberknife as a suitable modality for SBRT delivery.

Publications which were followed-up long enough to estimate the 5-year bRFS of radical prostatectomy (RP), normofractionated external beam radiation therapy (EBRT), and hypofractionated Cyberknife SBRT are summarized in Table 4. The 5-year bRFS of RP was 82% in Bianco et al^12^, 88% in Drouin et al^13^, and 76% in Amling et al.^14^ Results of EBRT were comparable to RP when the doses were high enough (≥70 Gy).^15,16^ SBRT achieved favorable outcomes (5-year bRFS of 74.1–97%) compared to RP or EBRT, even though the number of patients was small.^4,5^ In our analysis, the grade 3 or higher late urinary and rectal toxicity was 4.4% and 0%, respectively. Our toxicity profile is comparable to that of Katz et al^5^ which reported that 2% of late grade 3 genitourinary toxicity but no late grade 3 or higher gastrointestinal toxicity. The effectiveness of SBRT for a total of 1100 patients with clinically localized prostate cancer was tested. PSA relapse-free survival rates after SBRT compared favorably with other definitive treatments for low and intermediate risk patients.^17^ CyberKnife SBRT produces excellent long-term biochemical control rates. Median PSA levels continue to compare favorably with other radiation modalities with 7-year follow up.^18^

**TABLE 4:**
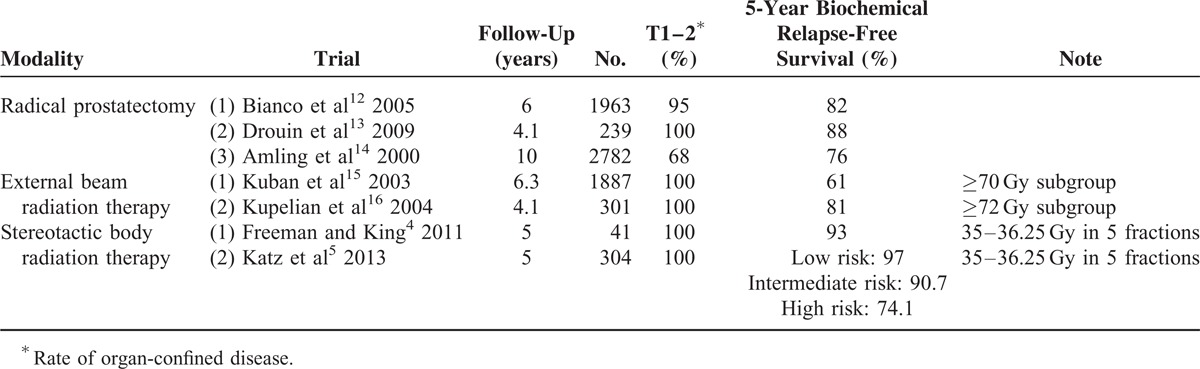
The Biochemical Relapse-Free Survival of Radical Prostatectomy, External Beam Radiation Therapy, and Stereotactic Body Radiation Therapy

The median age of our patients was 73 years. This is comparable to other contemporary Cyberknife SBRT studies along with those of Katz et al.^18^ and Bolzicco et al.^19^ However, Bolzicco et al had short median follow-up time (20 months), when compared with our follow-up time of 63 months. With cut-off eligibility of ECOG 0-1, their patients with old age had good performance status.^20^ There has been no current Cyberknife SBRT study that reported the comorbid conditions of patients in details as we analyzed in this study.

This study has several limitations. Our study had a small number of patient population. The confounding effect caused by hormonal therapy cannot be excluded.^21^ Heterogeneity of the patients better represents the actual efficacy of SBRT in everyday clinical setting but creates a dilemma that the analysis of the pure effect of SBRT may be disturbed. Nevertheless, we have a median follow-up of 63 months, which is longer compared with similar series of the contemporary. We not only included T3 patients and high-risk group, yet we also included patients with comorbidities, which might have contributed to the slightly lower 5-year bRFS of 89.7% compared with other studies.^4,5,22^ With more diverse and unfiltered patient pool, our results are less biased and more representative of the actual efficacy of SBRT.

## CONCLUSION

In an investigational analysis, SBRT using Cyberknife in prostate cancer patients with old age and comorbidities were favorable and safe in terms of survival and toxicity. Considering the long-term survival of prostate cancer patients and late occurrence of toxicity of the SBRT, longer follow-up and toxicity evaluation are necessary.
